# Mapping the research landscape of extrafloral nectaries: a comprehensive bibliometric analysis (1894–2026)

**DOI:** 10.3389/fpls.2026.1848739

**Published:** 2026-06-26

**Authors:** Akash Basnet, Nirmal Joshee

**Affiliations:** Department of Agricultural Sciences, College of Agriculture, Family Sciences, and Technology, Fort Valley State University, Fort Valley, GA, United States

**Keywords:** ant-plant interactions, bibliometric analysis, extrafloral nectaries, indirect plant defense, mutualism, nectar chemistry, PRISMA

## Abstract

Extrafloral nectaries (EFNs) are nectar-secreting glands on vegetative organs that mediate indirect plant defense by recruiting predatory arthropods. Documented in over 4,999 species across 129 families, with at least 457 independent evolutionary origins, they are the intersection of anatomy, chemical ecology, entomology, and evolutionary biology, yet no study has systematically mapped the bibliometric structure of this field. To the best of our knowledge, we present the first comprehensive science-mapping analysis of global EFN research. Bibliographic records were retrieved from Scopus and the Web of Science Core Collection, then merged, deduplicated, and screened in accordance with PRISMA 2020 guidelines adapted for bibliometric research. Quantitative analyses were performed using the R package bibliometrix (v5.2.1) and VOSviewer (v1.6.20). The final dataset comprised 1,279 publications (1894–2026) across 343 sources by 2,660 authors. A logistic growth model (R² = 0.938) indicated the field has reached approximately 87.6% of projected maturity, with peak growth around 2016. Brazil, the USA, and Mexico led corresponding-author output (44.5% combined), while African contributions remained marginal and Japanese and Indian contributions were substantial but internationally isolated. Oecologia headed fourteen core journals under Bradford’s Law, and Del-Claro K was the most prolific author. Walktrap clustering identified five thematic clusters: nectary anatomy/chemistry, EFN–ant ecological networks, biological control, mutualism–herbivory–indirect defense, and nectary ultrastructure. Thematic evolution traced an arc from morphological description through ecological experimentation to network-level analysis, with climate change emerging as a distinct theme in the most recent period (median keyword year: 2021). The biological control cluster remained the smallest and most isolated, despite demonstrated agricultural applications. International collaboration was concentrated along the Brazil–USA and Brazil–Mexico corridors across 262 bilateral links. These results provide a quantitative foundation for identifying knowledge gaps, particularly the underrepresentation of African and Asian research communities, the nascent climate-change frontier, and the disconnect between fundamental EFN ecology and applied pest management.

## 1 Introduction

**Figure 1:**
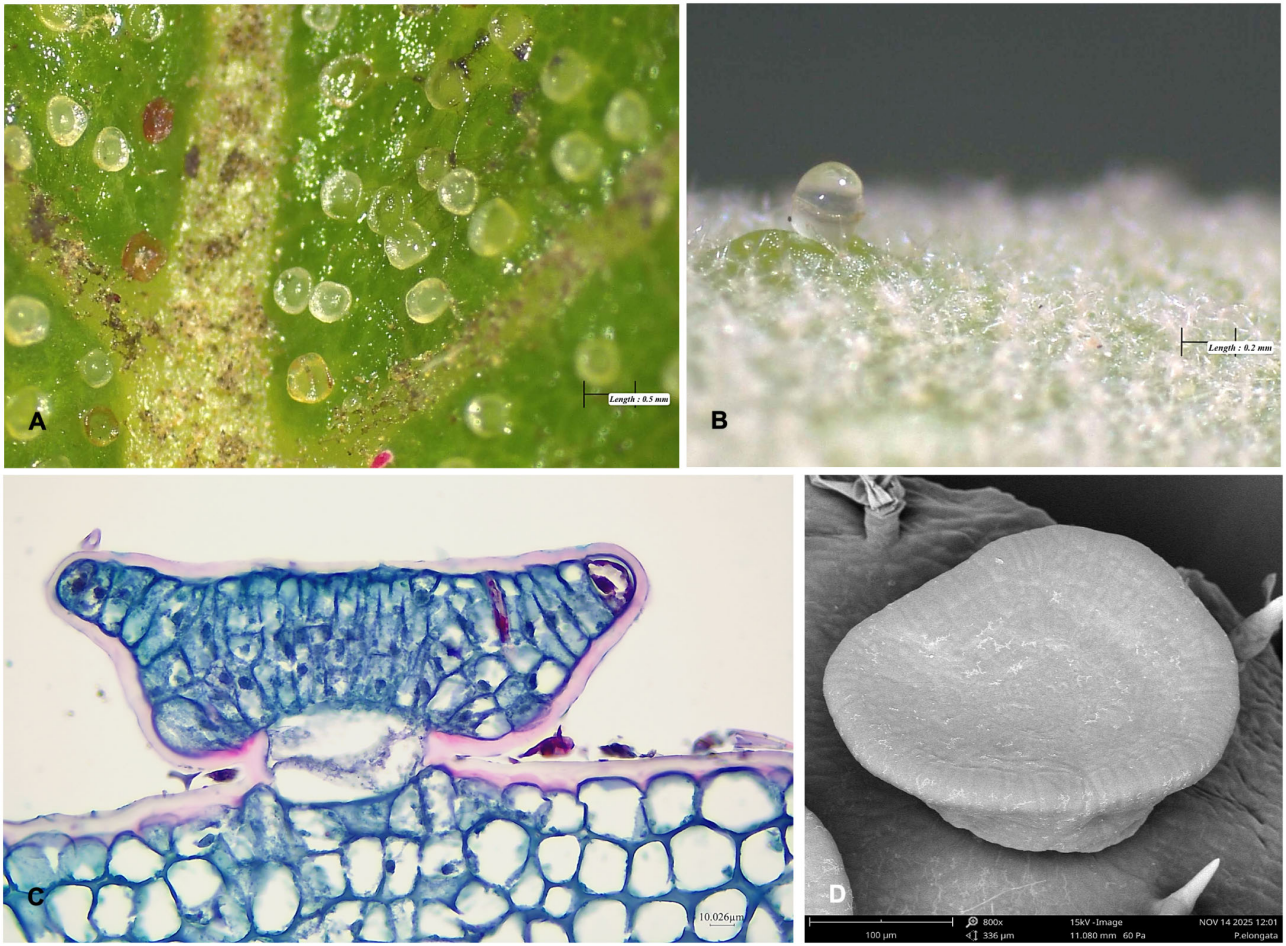
Extrafloral nectaries (EFNs) of *Paulownia fortunei*. **(A)** Stereomicroscopic image of the abaxial leaf surface showing EFNs distribution across the lamina; scale bar = 0.5 mm. **(B)** Close-up stereomicrograph of an EFN, showing the nectar accumulating at the top; scale bar = 0.2 mm. **(C)** Cross-section of an EFN stained with safranin and fast green, revealing the multicellular secretory head with densely staining cytoplasm, raised by a basal cell and underlying parenchymatous tissue; scale bar = 10.02 µm. **(D)** Scanning electron micrograph (SEM) of an EFN resembling a cup-shaped structure imaged at 800×; scale bar = 100 µm.

Plants have developed diverse defensive mechanisms against herbivory, which include physical barriers, such as thorns and trichomes, and chemical deterrents, such as alkaloids and terpenoids. They also use indirect strategies, recruiting other organisms as protective agents (Price et al., 1980; Heil and McKey, 2003). Among these indirect defenses, extrafloral nectaries (EFNs) are glandular structures that secrete sugary fluid on vegetative and reproductive organs outside the flower (Bentley, 1977; Koptur, 1992; Marazzi et al., 2013). Morphologically, EFNs exhibit wide structural diversity. Zimmermann (1932) proposed one of the earliest classification systems, categorizing EFNs into four types: formless nectaries, which lack defined anatomical boundaries; flattened nectaries, consisting of a secretory epidermis integrated with the organ surface; pit nectaries, which are sunken into the tissue; and elevated nectaries, which protrude above the surface as dome-shaped or capitate glands with a distinct secretory epidermis, sub-glandular parenchyma, and vascular connection. The EFNs of *Paulownia fortunei* illustrated in (**Figure 1**) are representative of the elevated type, exhibiting the characteristic dome-shaped secretory head, basal cell, and underlying parenchymatous tissue described above. Predatory arthropods, mainly ants, are attracted by the concentrated carbohydrate reward. While feeding on the secretion, they patrol the plants and deter herbivorous insects. This exchange of nutrition and protection is a well-studied example of mutualism in terrestrial ecosystems (Rico-Gray and Oliveira, 2007). A recent global compilation documented 4,766 records of ants feeding on EFNs from 342 published studies (1941–February 2024), spanning 40 countries, 441 plant species across 65 families, and 519 ant species across 7 subfamilies, with Brazil and Mexico contributing the largest share of records and Fabaceae and *Camponotus* emerging as the most frequently recorded plant family and ant genus, respectively (Novais et al., 2025). There is a wide taxonomic diversity among EFN-bearing plants. Weber and Keeler documented 135 years of observational records of EFNs and compiled a database, the World List of Plants with Extrafloral Nectaries (Keeler et al., 2026). As of now, EFNs are catalogued over 4,999 species distributed over 982 genera and 129 families.^1^ Phylogenetic studies suggest EFNs arose independently in no fewer than 457 lineages (Marazzi et al., 2013; Weber and Agrawal, 2014). This degree of convergent evolution underscores the strong fitness advantages conferred by indirect defense via nutritional rewards.

Tracing back to a historical standpoint, EFNs’ research originates from the late nineteenth century, with the earliest recorded contribution in the Annals of Botany in 1894. Research progressed through distinct phases. The concept of myrmecophily was introduced by Delpino (1886). Janzen (1966) provided the first demonstration that ant occupants reduce herbivore damage through experimental work on *Acacia*–ant mutualisms in Central America. Later, the conceptual framework of indirect defense was developed by Bentley (1977). An experiment demonstrating that the extrafloral nectar secretion is regulated by the jasmonic acid signaling pathway and can be induced by leaf damage establishes EFNs as dynamic, plastic defense organs rather than static morphological features, and is a molecular turning point in EFNs research (Heil et al., 2001). Today, research on EFNs extends over proteomics, ecological network theory, climate-change biology, and agroecological applications.

Quantitative meta-analyses have reported that the presence of ants on EFN-bearing plants reduces herbivore damage by 50–60% on average and increases reproductive output by roughly 40% (Rosumek et al., 2009; Trager et al., 2010). Secretion released by EFNs is composed of a mixture of sucrose, glucose, and fructose with variable amino-acid profiles, lipids, and secondary metabolites that modulate the identity and behavior of visiting arthropods, creating a unique chemical ecology mediating defense (Heil, 2011, Heil, 2015). Phylogenetic analyses suggest the presence of EFN correlates with higher speciation rates, suggesting that the trait itself may promote lineage diversification (Weber and Agrawal, 2014).

Although EFN research is diversified and continuously expanding, no study has systematically analyzed the bibliometric structure of EFN research as a distinct domain. Bibliometric analysis applies statistical methods to publication and citation data to reveal patterns in scientific output, identify influential research and contributors, detect thematic clusters, and trace intellectual orientation (Donthu et al., 2021; Zupic and Čater, 2015). Such analyses have been applied for research in plant responses to drought and heat (Cui et al., 2022), ant-plant interaction networks (Juárez‐Juárez et al., 2023), and plant defense mutualisms broadly, but the specific EFN literature, which is the hub of anatomy, chemical ecology, entomology, and evolutionary biology, has not been studied in detail.

The current study addresses this knowledge gap by conducting the first comprehensive bibliometric analysis of global research on EFNs. Following PRISMA 2020 guidelines adapted for bibliometric studies (Page et al., 2021) and the analytical framework developed by Donthu et al. (2021), we systematically analyze 1,279 documents spanning 132 years (1894–2026). The objectives of our study are (i) to characterize the publication growth trends and model the research life cycle; (ii) to identify the most productive and impactful countries, institutions, authors, and journals; (iii) to map the conceptual structure of the field through keyword co-occurrence and thematic analysis; (iv) to trace the thematic evolution across four temporal periods; and (v) to analyze international collaboration patterns and identify underexplored research frontiers.

## 2 Materials and methods

This study was designed following the PRISMA 2020 reporting guidelines for systematic reviews (Page et al., 2021) and the methodological recommendations proposed by Donthu et al. (2021) and Aria and Cuccurullo (2017). The overall workflow comprised five sequential phases: (1) research design and search strategy formulation, (2) systematic data retrieval, (3) data processing and quality control, (4) bibliometric analysis, and (5) visualization and interpretation.

### 2.1 Database and search strategy

Bibliographic data were retrieved from two major multidisciplinary databases: the Web of Science (WoS) Core Collection (Clarivate) and Scopus (Elsevier). The selection of two databases ensures a comprehensive collection of the EFN database, as WoS and Scopus differ in their journal indexing scope, with WoS and Scopus respectively covering approximately 22,000 and 27,000 journals (Mongeon and Paul-Hus, 2016; Singh et al., 2021).

The search was conducted in February 2026 using the Advanced Search interface of both databases. The following Boolean string was applied: (“extrafloral nectar*” OR “extra-floral nectar*” OR “extra floral nectar*” OR “extranuptial nectar*”) OR ((“EFN” OR “EFNs”) AND (“nectar*” OR “ant*” OR “insect*” OR “mutualism”)). In Scopus, this string was applied within the TITLE-ABS-KEY fields, while in Web of Science, the TS (Topic Search) field was used.

The acronym “EFN” was combined with ecology-related terms to exclude records where EFN refers to non-botanical meanings (e.g., European Financial Network). No restrictions were imposed on publication year, language, or document type at the search stage, to capture the broadest possible set of records for subsequent screening.

### 2.2 Eligibility criteria and screening

**Figure 2:**
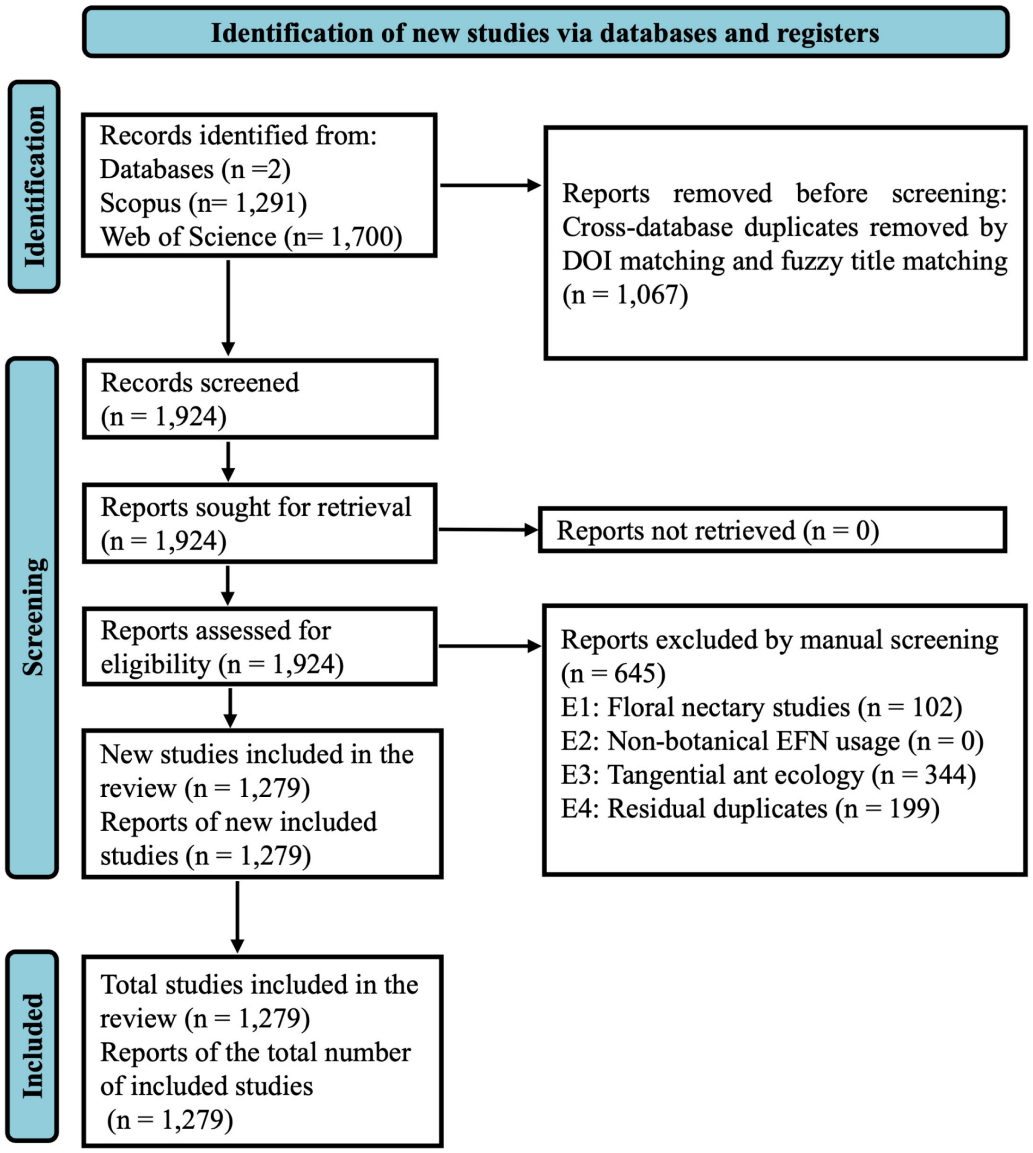
PRISMA 2020 flow diagram adapted for the bibliometric analysis of extrafloral nectary research. Records identified from Scopus (n = 1,291) and Web of Science (n = 1,700) duplicates removed across merged database (n = 1,067) and manual screening (n = 645), relevance screening by keyword matching across abstracts, titles, and keywords, final dataset for analysis (n = 1,279).

Records from both databases were exported with all available metadata, including citation details, bibliographical data, abstracts, author and index keywords, affiliations, and funding information. The screening and selection process followed the PRISMA 2020 flow diagram (**Figure 2**). The following criteria were established to ensure core data collection. Inclusion criteria: (a) the document was indexed in the Scopus and WoS databases with complete bibliographic metadata; (b) the abstract, title, or author keywords contained at least one direct reference to extrafloral nectaries using terms such as “extrafloral nectar,” “extrafloral nectary,” “extrafloral nectaries,” “extra-floral nectar,” “extra-floral nectary,” “extra-floral nectaries,” “extranuptial nectar,” “extranuptial nectary,” “extranuptial nectaries,” “foliar nectary,” “foliar nectaries,” or the abbreviation “EFN”/”EFNs”; (c) there were no restrictions on language, publication year, or document type. Exclusion criteria: (a) records focused solely on floral nectaries, pollination biology, or general ant ecology without relevant EFN content; (b) duplicate records identified through title matching. Exclusion criteria were applied in priority order: (E1) Floral-nectary-only studies addressing floral nectaries, pollination biology, or floral nectar chemistry (e.g., pollination biology of *Arabidopsis* or *Nicotiana*; SWEET9 or CRABS CLAW pathway studies on floral systems alone); (E2) Non-botanical use of the “EFN” acronym (e.g., European Financial Network, Effective Number of Females; (E3) Tangential ant ecology, where the record concerned ant biology, ant taxonomy, or ant–plant interactions in which EFNs were not the focus; and (E4) Residual within-database duplicates not captured by automated cross-database deduplication. All flagged records were manually screened against criteria E1–E4, with the original full text when the abstract was insufficient. The complete record-by-record screening decisions for all 1,924 unique records, including the assigned exclusion code (E1, E3, or E4) for each excluded record, are deposited as a CSV file in the public repository, so that the screening procedure can be independently audited.

### 2.3 Data cleaning and deduplication

Documents from both databases were merged. Duplicate removal was performed using the duplicatedMatching() function in the bibliometrix R package version 5.2.1 with the restricted Damerau-Levenshtein distance (tolerance threshold = 0.95). Relevance screening employed an automated keyword-matching algorithm that searched the combined text of abstracts, titles, author keywords, and index keywords for a comprehensive set of EFN-related terms (including spelling variants and synonyms). Records failing to match any EFN term were flagged, manually verified, and excluded if confirmed as irrelevant. The complete PRISMA flow diagram with record counts at each stage (**Figure 2**).

### 2.4 Bibliometric analysis tools

#### 2.4.1 R-based bibliometrix package

The primary analytical platform was the bibliometrix R package version 5.2.1 (Aria and Cuccurullo, 2017) accessed through its Shiny web interface, Biblioshiny. Data were imported via the convert2df() function (dbsource = ‘scopus’, format = ‘csv’) for Scopus, (dbsource = ‘Web of Science’, format = ‘plaintext’) for WoS. The resulting bibliographic data frames were then merged into a single dataset using mergeDbSources() with duplicate removal enabled. The following analyses were performed: (a) Descriptive statistics: annual publication trends, most productive sources/authors/countries/institutions/document-type distribution, and collaboration metrics. (b) performance indicators, Bradford’s Law of journal scattering, Lotka’s Law of author productivity, and h-index calculations for sources and authors within the dataset; (c) conceptual structure, keyword co-occurrence network (author keywords, Walktrap clustering, Fruchterman-Reingold layout), Callon’s thematic map (centrality–density strategic diagram), thematic evolution (four temporal periods: 1894–1990, 1991–2005, 2006–2015, 2016–2026), Multiple Correspondence Analysis (MCA), and three-field Sankey plots; (d) social structure, co-authorship networks at author, institutional, and country levels, single-country vs. multiple-country publication (SCP/MCP) ratios, and collaboration world-map visualization; (e) life-cycle modeling, logistic growth-curve fitting to cumulative annual production data (parameters K, t_m_, Δt). All author- and source-level impact metrics reported in this study (h-index, m-index, total citations) are local metrics computed within the 1,279-document EFN corpus using the Hindex() function of the bibliometrix R package (accessed via the Biblioshiny interface). They quantify intra-field citation impact on EFN research specifically and should not be interpreted as career-wide bibliometric indicators.

Publication-quality figures were generated using Python (version 3.12) with matplotlib, plotly, and networkx libraries, based on the bibliometrix-processed dataset.

#### 2.4.2 VOSviewer

VOSviewer (version 1.6.20: Van Eck and Waltman, 2010), developed at Leiden University, was used for complementary network visualizations. Keyword co-occurrence maps, co-authorship networks, and density overlays were generated using the VOS mapping algorithm and clustering technique. VOSviewer was primarily chosen for its ability to produce high-resolution, publication-quality overlay and density maps that complement Bibliometrix’s clustering output.

### 2.5 Life-cycle analysis

The maturity of the EFN research field was assessed by fitting a logistic growth model to annual publication series (1894–2026). Fitting was performed on the annual (rate) series rather than the cumulative series to avoid the inflated goodness-of-fit and biased asymptote estimates that arise from autocorrelated residuals in cumulative data. The model estimates three parameters: the saturation points K (the projected total number of publications when growth ceases), the inflection year t_m_ (the year with the maximum annual growth rate), and the growth duration Δt (the period encompassing most of the growth phase). The R² value indicates goodness of fit. Cumulative trajectories and milestone years at 10%, 50%, 90%, and 99% of K were derived analytically from the fitted parameters model.

## 3 Results and discussion

### 3.1 Overview of the bibliographic collection

From the initial databases, 2,991 records (1,291 from Scopus and 1,700 from Web of Science) were retrieved. Using cross-database deduplication, 1,067 overlapping records were removed, leaving 1,924 unique documents. Manual screening excluded 645 records that did not relate to extrafloral nectaries. After all the screening processes, a final working dataset was generated, consisting of 1,279 documents published across 343 sources between 1894 and 2026 (**Table 1**). Research articles constituted 91.5% of the collection (n = 1,170), followed by reviews (4.1%; n = 53), book chapters (2.0%; n = 25), conference papers (0.9%; n = 12), and other document types (1.5%). Altogether 2,660 authors contributed to the literature, producing 3,197 unique author keywords and 3,755 Keywords Plus terms. Single-authored documents numbered 167, with 127 unique single authors. Co-authorship averaged 3.46 authors per document, and 27.29% of all publications involved international collaboration (**Table 1**). The average citation count per document was 33.42, and the mean document age was 15.5 years.

**Table 1:** Main information about the EFN bibliometric collection.

Metric	Value
Timespan	1894–2026
Sources (journals, books, etc.)	343
Total documents	1,279
Annual growth rate	1.83%
Document average age	15.5 years
Average citations per document	33.42
Author keywords (DE)	3,197
Keywords Plus (ID)	3,755
Total authors	2,660
Authors of single-authored documents	127
Single-authored documents	167
Co-authors per document	3.46
International co-authorships	27.29%
Articles	1,170 (91.5%)
Reviews	53 (4.1%)
Book chapters	25 (2.0%)
Conference papers	12 (0.9%)

### 3.2 Annual publication trends and research life cycle

**Figure 3:**
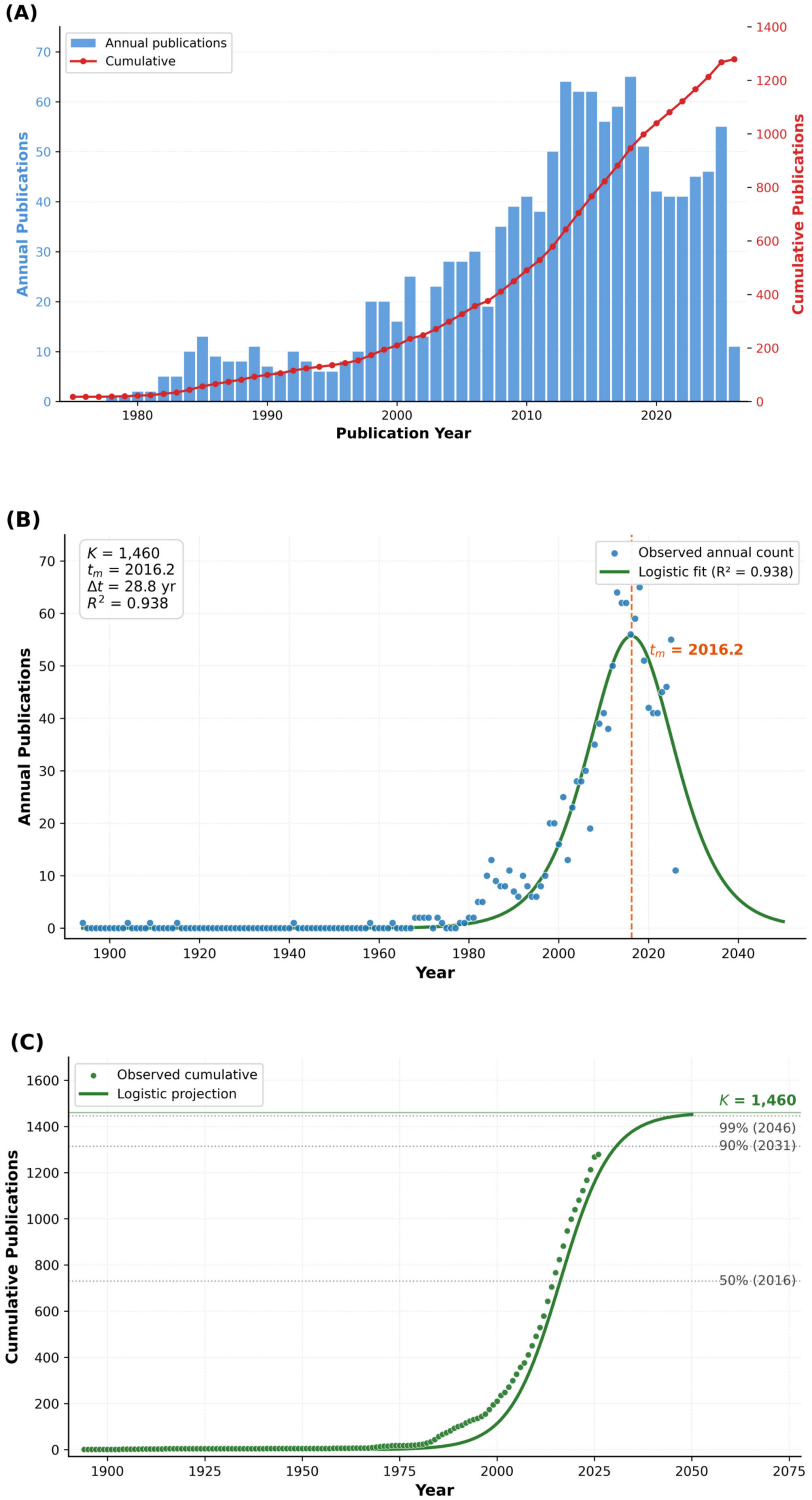
Publication trends and life-cycle analysis of EFN research. **(A)** Annual scientific production with cumulative trend (1894–2026); **(B)** Life-cycle logistic growth curve (K ≈ 1,460; R² = 0.938; t_m_ ≈ 2016.2); **(C)** Cumulative growth with milestone percentages (50%, 90% of K).

The earliest recorded EFN publication dates to 1894, yet annual output remained very low, rarely exceeding one to two papers per year until the late 1960s and 1970s, when the influence of Janzen’s (1966) ant–*Acacia* experiments and Bentley’s (1977) review began to attract wider attention (**Figure 3A**). A sustained increasing trend took hold from the early 1980s onward. Period-level tallies show the acceleration: 20 papers appeared before 1980 (~0.2/year), 174 during 1980–1999 (~8.7/year), 256 during 2000–2009 (~25.6/year), 548 during 2010–2019 (~54.8/year), and 281 from 2020 through early 2026 (~40.1/year, noting that 2026 data were incomplete at retrieval). The single most productive year was 2018, with 65 publications.

A logistic growth model fitted to the annual publication series yielded an R² of 0.938, indicating an excellent fit (**Figure 3B**). The estimated saturation point (K) was approximately 1,460 publications, with an inflection year (t_m_) of 2016.2 and a growth duration (Δt) of 28.8 years. At the time of data retrieval, cumulative output stood at 1,279 documents, approximately 87.6% of model-projected saturation under the assumption that the underlying growth process is logistic. This estimate should be interpreted with caution: a logistic model fitted to a still-active research field is necessarily an extrapolation, and its parameters are sensitive to the most recent years, which are themselves incompletely indexed. The estimate is best read as an indication that the rate of new publication has decelerated relative to the 2000–2018 expansion phase, not as evidence that the field has stopped growing. Milestone projections place the 90% mark at approximately 2031 and the 99% mark near 2046 (**Figure 3C**). The model-predicted peak annual output of approximately 56 papers occurred in 2016 (= t_m_), close to but slightly earlier than the observed empirical peak of 65 papers in 2018. These metrics indicate that the established themes of EFN research anatomical description, ant–plant ecology in Neotropical biomes, and Bentley-style indirect-defense experiments have entered a maturity phase, with cumulative output approaching the model-projected asymptote. The field, however, is unlikely to be terminally saturated. Two emerging frontiers are poised to generate a secondary growth phase not captured by the present logistic fit: the effects of climate change on EFN-mediated mutualisms (Calixto et al., 2021; do Rosario Nogueira, 2025), and the application of single-cell transcriptomics and CRISPR-based functional genomics to nectary development (building on the floral-nectary work of Lin et al., 2014, and Lee et al., 2005). Because these represent new research themes superimposed on the existing trajectory rather than a continuation of it, the most appropriate interpretation is that the classical EFN research programme has matured while the field is poised to enter a methodologically distinct second wave.

These metrics indicate that EFN research has entered a maturity phase; however, emerging connections to climate change and advanced molecular techniques (CRISPR, single-cell transcriptomics) may extend the growth curve beyond the logistic prediction.

### 3.3 Most productive sources and Bradford’s law

**Figure 4:**
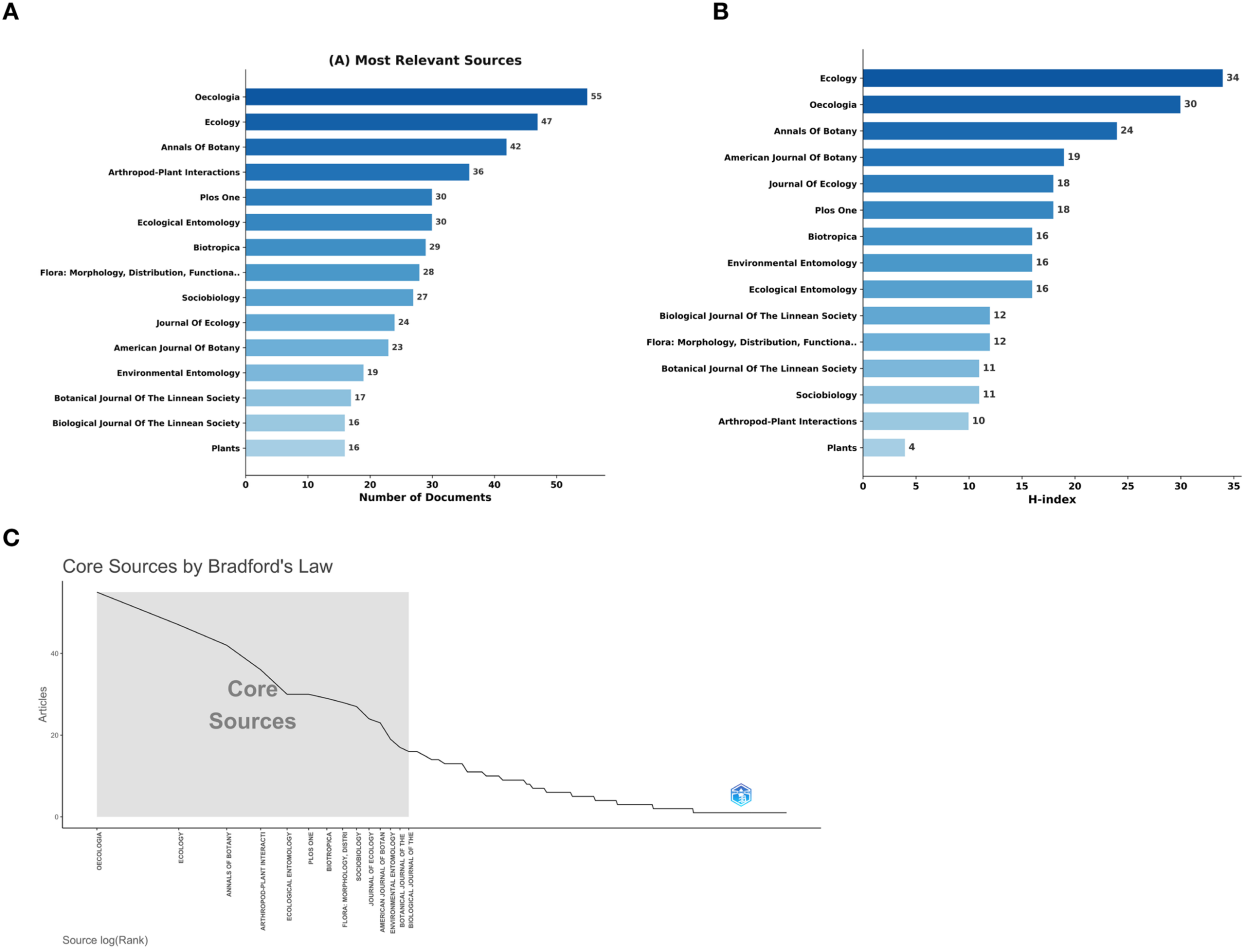
Source analysis. **(A)** Top 15 most relevant journals by publication count; **(B)** h-index of the top 16 journals publishing extrafloral nectary research; **(C)** Bradford’s Law core sources (Zone 1: 14 journals).

The 1,279 documents were distributed across 343 sources. Oecologia published the greatest number of EFN papers (55), followed by Ecology (47), Annals of Botany (42), Arthropod-Plant Interactions (36), Ecological Entomology (30), PLoS ONE (30), Biotropica (29), Flora: Morphology, Distribution, Functional Ecology of Plants (28), Sociobiology (27), and Journal of Ecology (24) (**Figure 4A**). Within the dataset, Ecology achieved the highest local h-index (34), indicating that 34 of its EFN papers had each received at least 34 citations within the collection. Oecologia followed with an h-index of 30, and Annals of Botany with 24 (**Figure 4B**).

Bradford’s Law divided the 343 sources into three zones of roughly equal cumulative output (**Figure 4C**). Zone 1 (core) included 14 journals that account for approximately one-third of all publications, spanning from Oecologia (55 articles) to Biological Journal of the Linnean Society (16 articles). The remaining two-thirds of publications were scattered across 329 journals in Zones 2 and 3, demonstrating the classic concentration, then dispersion pattern of scientific literature.

### 3.4 Most productive and impactful authors

**Figure 5:**
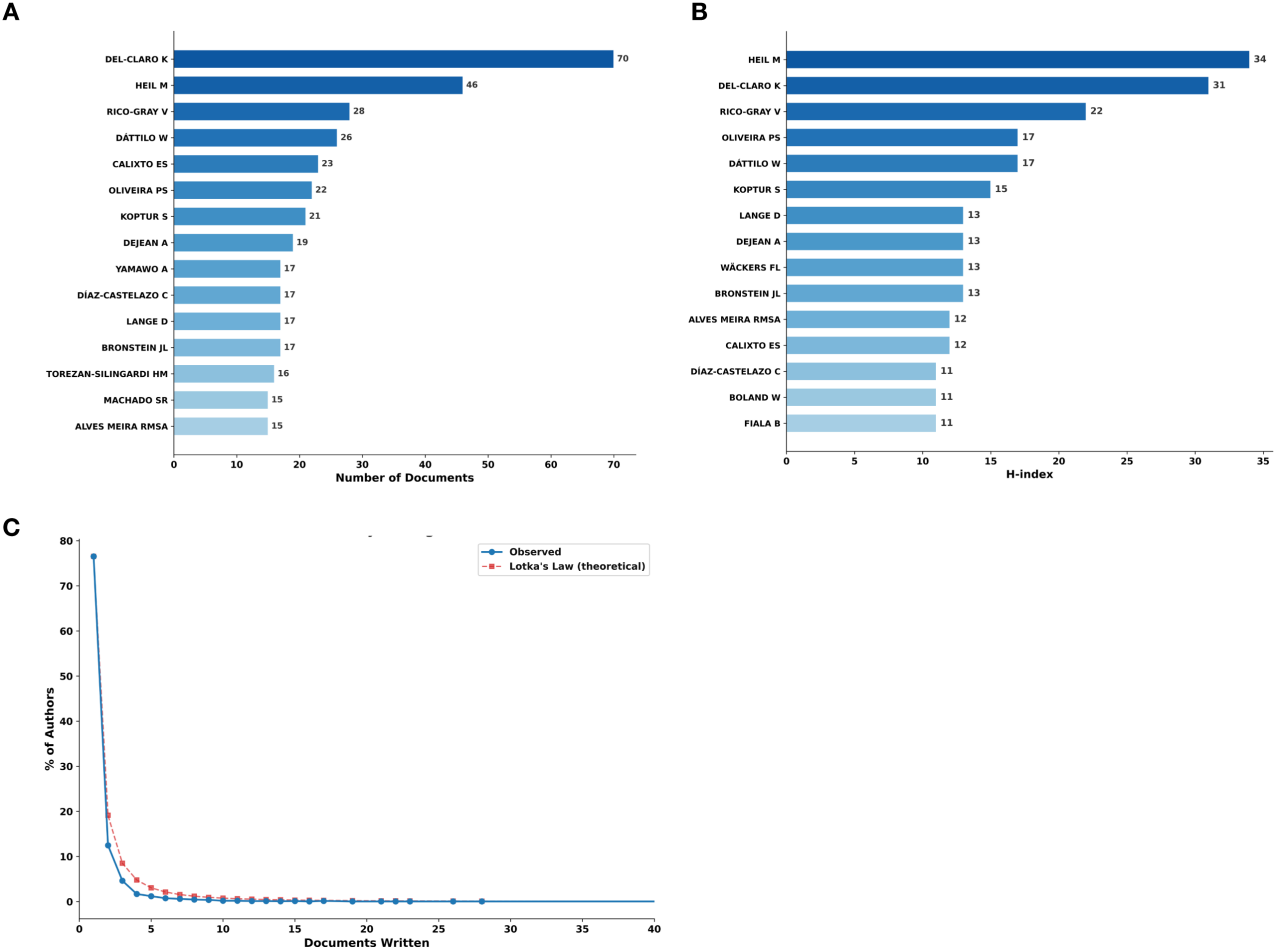
Author analysis. **(A)** Top 15 most productive authors; **(B)** Author local impact by h-index computed within the 1,279-document EFN corpus; **(C)** Lotka’s Law: observed vs. theoretical author-productivity distribution.

A total of 2,660 authors contributed to the EFN research. Del-Claro K was the most prolific contributor with 70 full-count publications (fractionalized: 22.7), followed by Heil M (46), Rico-Gray V (28), Dáttilo W (26), Calixto ES (23), Oliveira PS (22), Koptur S (21), Dejean A (19), Bronstein JL (17), Díaz-Castelazo C (17), Lange D (17), and Yamawo A (17) (**Figure 5A**).

When ranked by h-index within the EFN collection, Heil M led with 34, reflecting the outsized citation impact of his work on jasmonic-acid signaling, volatile-mediated defense, and nectar chemistry (5,176 total citations). Del-Claro K followed (h = 31; 2,588 citations), then Rico-Gray V (h = 22; 1,803 citations), Dáttilo W (h = 17; 1,011), and Oliveira PS (h = 17; 1,163). The m-index—h normalized by career length—highlighted Heil M (1.26) and Dáttilo W (1.13) as the researchers with the most consistent annual impact (**Figure 5B**).

The production-over-time plot revealed generational patterns (**Supplementaryry Figure 1**). Koptur S (active since 1984) and Oliveira PS (since 1987) represent the pioneering generation. Del-Claro K (since 1996) and Heil M (since 2000) led the field’s most productive phase. Among the newest cohort, Calixto ES (since 2015; h = 12; m = 1.0) stands out for rapid impact accumulation.

Author productivity deviated from Lotka’s inverse-square law. Single-paper contributors made up 76.5% of all authors (2,036 of 2,660), surpassing the expected 62.5%, while two-paper authors (12.5%) fell below the predicted 15.6%. At the high end of the distribution, highly prolific researchers were more numerous than the model predicts (**Figure 5C**). These patterns show a two-tier structure: a wide group of researchers who encounter EFNs through other disciplines and a small but core group who sustain the field’s intellectual growth.

### 3.5 Leading countries, institutions, and international collaboration

**Figure 6:**
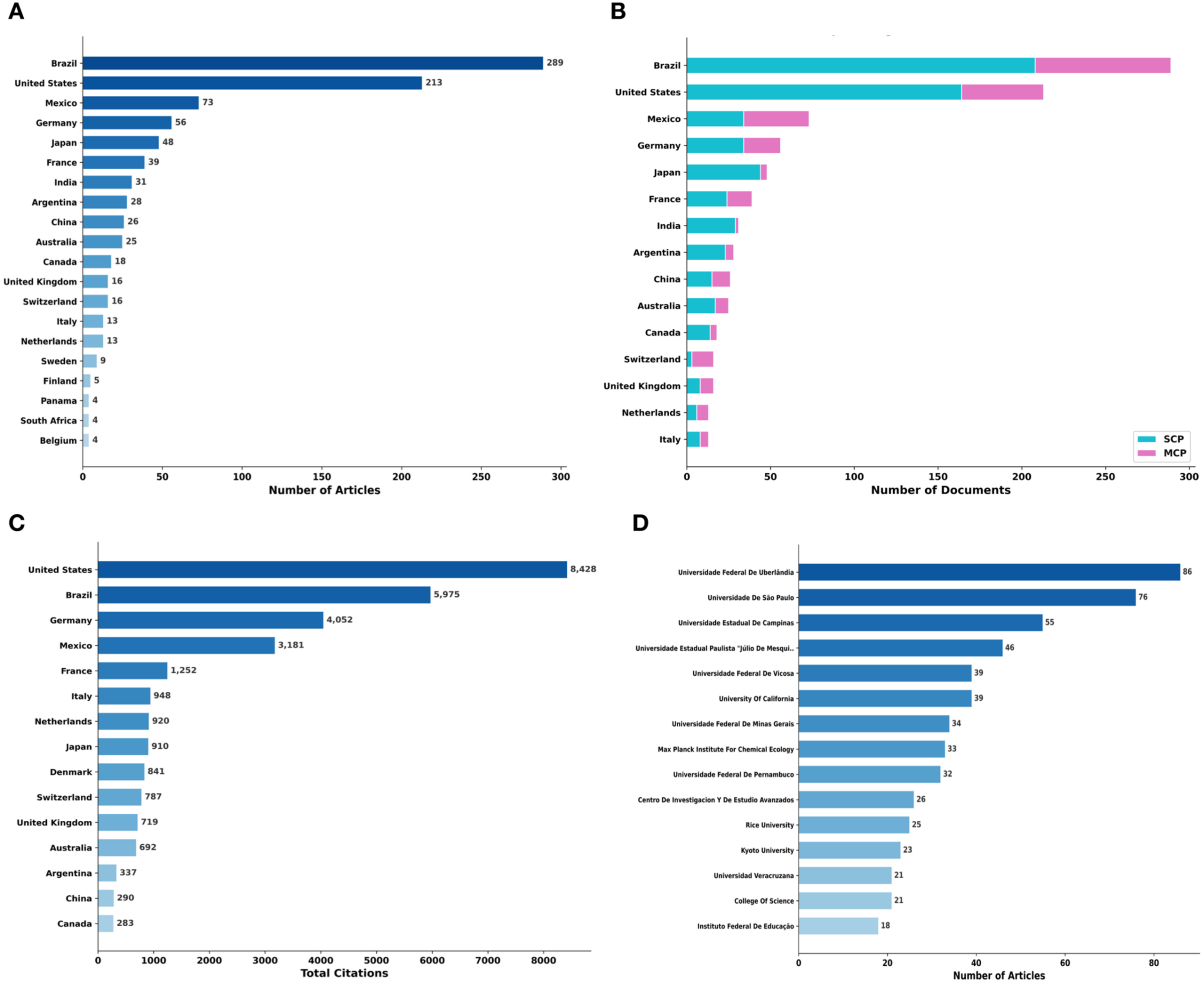
Geographic analysis. **(A)** Country scientific production (top 20); **(B)** Corresponding author’s countries with SCP/MCP breakdown; **(C)** Most cited countries (total citations); **(D)** Most relevant affiliations (top 15 institutions).

Corresponding-author analysis identified 51 countries. Brazil dominated with 288 articles (22.5%), followed by the USA (213, 16.7%), Mexico (73, 5.57%) (**Figure 6A**). The three leading nations together accounted for 45.0% of the total, illustrating a pronounced Neotropical concentration that mirrors the biogeographic hotspot of EFN diversity in the Cerrado savanna, Atlantic Forest, and Mexican tropical deciduous forests. This Neotropical focus reveals a knowledge gap. Africa, Southeast Asia, the Indian subcontinent, and the temperate zones of East Asia remain underrepresented in EFN research despite having diverse EFN-bearing floras. Japan (48 articles, MCP = 6.3%) and India (31 articles, MCP = 6.5%); these metrics show that they have large domestic research communities with minimal international collaboration (**Figure 6B**). Increasing cross-continental partnerships, particularly with African nations, where a collaboration network (49 bilateral links) involving 10 countries is marginal in comparison to their biodiversity. New cross-continental corridors would extend the taxonomic, biogeographic, and climatic range of EFN studies (**Supplementary Figure 2**).

The USA stands on top with a total of 8,318 citations according to total citation analysis, followed by Brazil (5,974) and Germany (4,052). But when we normalize by article count (dividing by the number of articles), smaller contributors show larger impact: Denmark averaged 210.2 citations per article, Italy 72.9, and Germany 72.4 (**Figure 6C**), suggesting that while Brazil and the USA dominate in output volume, some European groups have produced exceptionally highly cited individual contributions.

Mexico displayed the highest multiple-country publication ratio among major producers (MCP = 52.9%), reflecting its role as a geographic and intellectual bridge between North American and Latin American EFN research communities. Switzerland (81.3%) and the Netherlands (53.8%) were also highly internationalized, whereas Japan (6.3%) and India (6.5%) conducted EFN research almost entirely through domestic partnerships. The collaboration world-map recorded 262 unique bilateral links; the strongest corridors were Brazil–USA (56 joint papers), Brazil–Mexico (33), USA–Mexico (23), Brazil–Spain (22), and Mexico–Germany (19) (**Figure 6B**). This Neotropical concentration, while biologically justified, means that African and Asian contributions remain marginal relative to regional EFN-bearing floral diversity. The strongest Africa-involving corridor was USA–Kenya (7 joint papers), and France served as the primary European partner for Francophone West Africa (France–Cameroon: 3; France–Senegal: 2). Fostering stronger partnerships would diversify the taxonomic and climatic range of future work (**Supplementary Figure 2**).

Institutional-level visualization reveals that the Brazilian universities dominated the top rankings. The Universidade Federal de Uberlândia led with 86 publications, followed by the Universidade de São Paulo (76), Universidade Estadual de Campinas (55), Universidade Estadual Paulista (46), and Universidade Federal de Viçosa (39). The first non-Brazilian institution was the University of California (39), followed by the Max Planck Institute for Chemical Ecology (33)—the latter reflecting the prolific output of Heil M and collaborators on the molecular ecology of EFN-mediated interactions (**Figure 6D**).

### 3.6 Most globally cited documents

Fürstenberg-Hägg et al. (2013), a review of plant defense mechanisms and secondary metabolites published in the International Journal of Molecular Sciences, is on top with 747 total citations (53.4 per year). Three out of the five most cited works were authored or co-authored by Heil M, underscoring his foundational influence on modern EFN science through discoveries related to jasmonic-acid-induced nectar secretion (Heil and Silva Bueno, 2007), volatile-mediated indirect defense (Heil, 2008), and the ecological costs of nectar production (**Table 2**).

**Table 2:** Top 5 most globally cited documents in the EFN dataset.

Rank	Paper	Total citations	TC/Year
1	Fürstenberg-Hägg J, 2013, Int J Mol Sci	747	53.4
2	Heil M, 2008, New Phytol	624	32.8
3	Heil M, 2007, PNAS	585	29.3
4	Heil M, 2011, Trends Plant Sci	446	27.9
5	Arimura G-I, 2005, BBA-Mol Cell Biol Lip	428	19.5

The Meta-analysis by Rosumek et al. (2009), which quantified the results of ants on EFN-bearing plants across 106 studies, ranked seventh and remains the most cited primary-research synthesis in the field. Book chapter by Koptur’s (1992) in Insect-Plant Interactions ranked eighth with 335 citations, indicating the enduring influence of comprehensive treatments of EFN ecology.

### 3.7 Keyword analysis and thematic clusters

**Figure 7:**
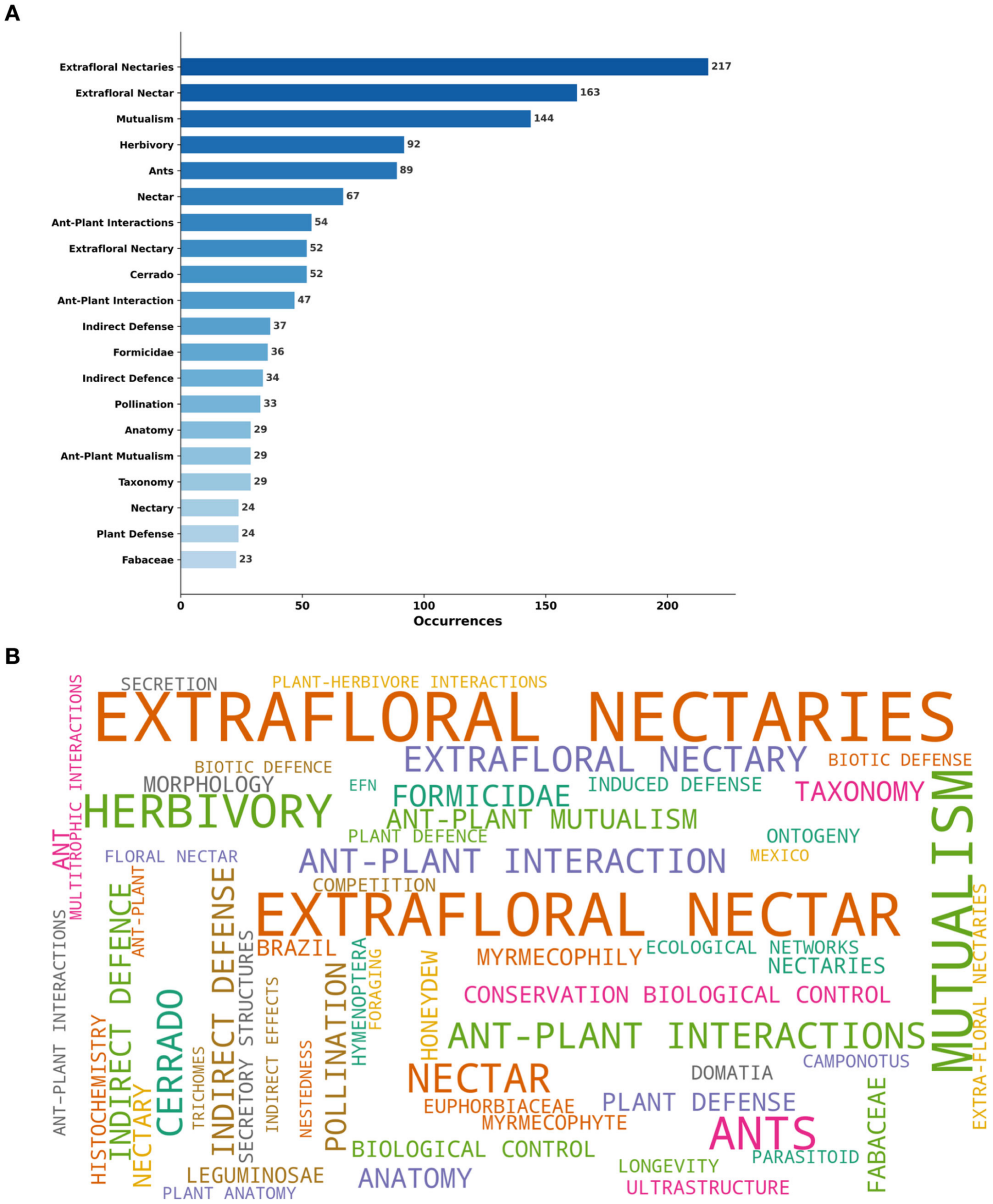
Keyword analysis. **(A)** Most frequent author keywords (top 20); **(B)** Word cloud of author keywords.

The most Frequently used author keywords were “extrafloral nectaries” (217 occurrences), “extrafloral nectar” (163), “mutualism” (145), “herbivory” (92), “ants” (89), “nectar” (67), “ant-plant interactions” (54), “cerrado” (52), “extrafloral nectary” (52), and “ant-plant interaction” (47). (**Figures 7A, B**).

**Figure 8:**
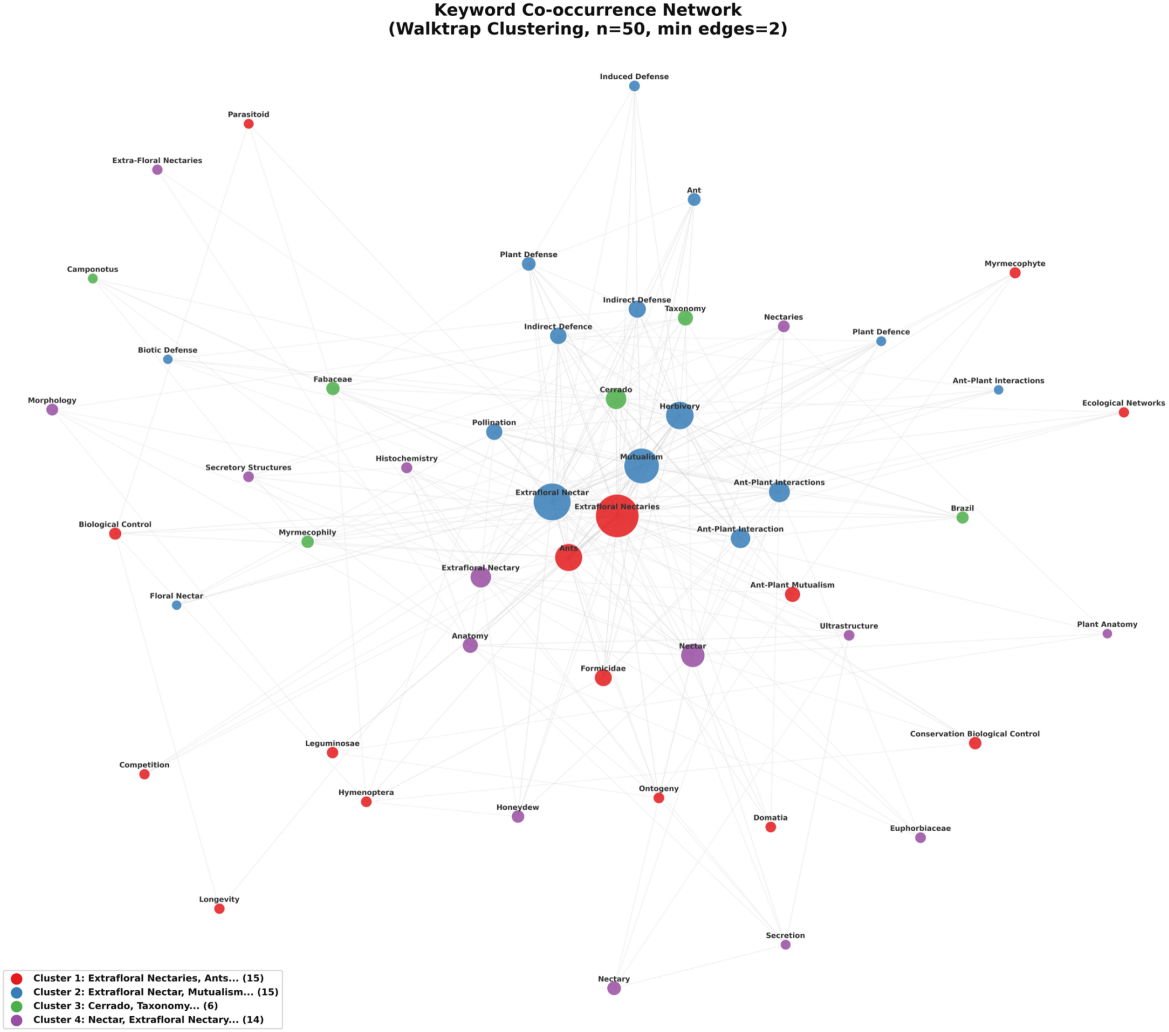
Keyword co-occurrence network generated using the Walktrap clustering algorithm (Fruchterman-Reingold layout, n = 50 nodes, minimum edges = 2). Node size is proportional to keyword frequency; node color represents cluster membership. Five clusters identified: nectary anatomy/chemistry (Cluster 1), EFN–ant ecological networks (Cluster 2), biological control (Cluster 3), mutualism–herbivory–indirect defense (Cluster 4), and nectary ultrastructure (Cluster 5).

Co-occurrence network analysis was done using the Walktrap clustering algorithm, which shows 50 keyword nodes arranged into five clusters (**Figure 8**).

Cluster 1 (11 nodes), Nectary anatomy and chemistry: nectar, extrafloral nectary, anatomy, morphology, nectaries, Leguminosae, histochemistry, Euphorbiaceae. This cluster captures trends in morphological characterization, phytochemical analysis, and plant-family associations.

Cluster 2 (11 nodes), EFN–ant ecological networks: extrafloral nectaries, ants, ant-plant interactions, ant-plant mutualism, taxonomy, Fabaceae, conservation, biological control, honeydew. As the largest and most central cluster, it represents the core ecological paradigm linking EFNs with arthropod-mediated protection.

Cluster 3 (3 nodes), Biological control: biological control, longevity, parasitoid. Compact cluster reflecting the applied agricultural area of EFN research in pest management.

Cluster 4 (22 nodes), Mutualism, herbivory, and indirect defense: extrafloral nectar, mutualism, herbivory, cerrado, ant-plant interaction, indirect defense, Formicidae, indirect defense. This large cluster encompasses the tri-trophic-interaction theoretical framework, with particular emphasis on the Brazilian Cerrado as a model system.

Cluster 5 (3 nodes), Nectary ultrastructure and secretion: nectary, ultrastructure, secretion. This cluster represents research on cellular structure and secretion mechanisms. Among the five clusters, Clusters 2 and 4 were the largest, indicating that the most EFN research focused on either ecological interactions at the community level or on how plants use nectar to defend against herbivores. Cluster 3 (applied entomology) signals an unexplored area. EFN-bearing cover crops have shown potential for pest suppression in agroecosystems, yet the research is limited.

### 3.8 Thematic map (strategic diagram)

**Figure 9:**
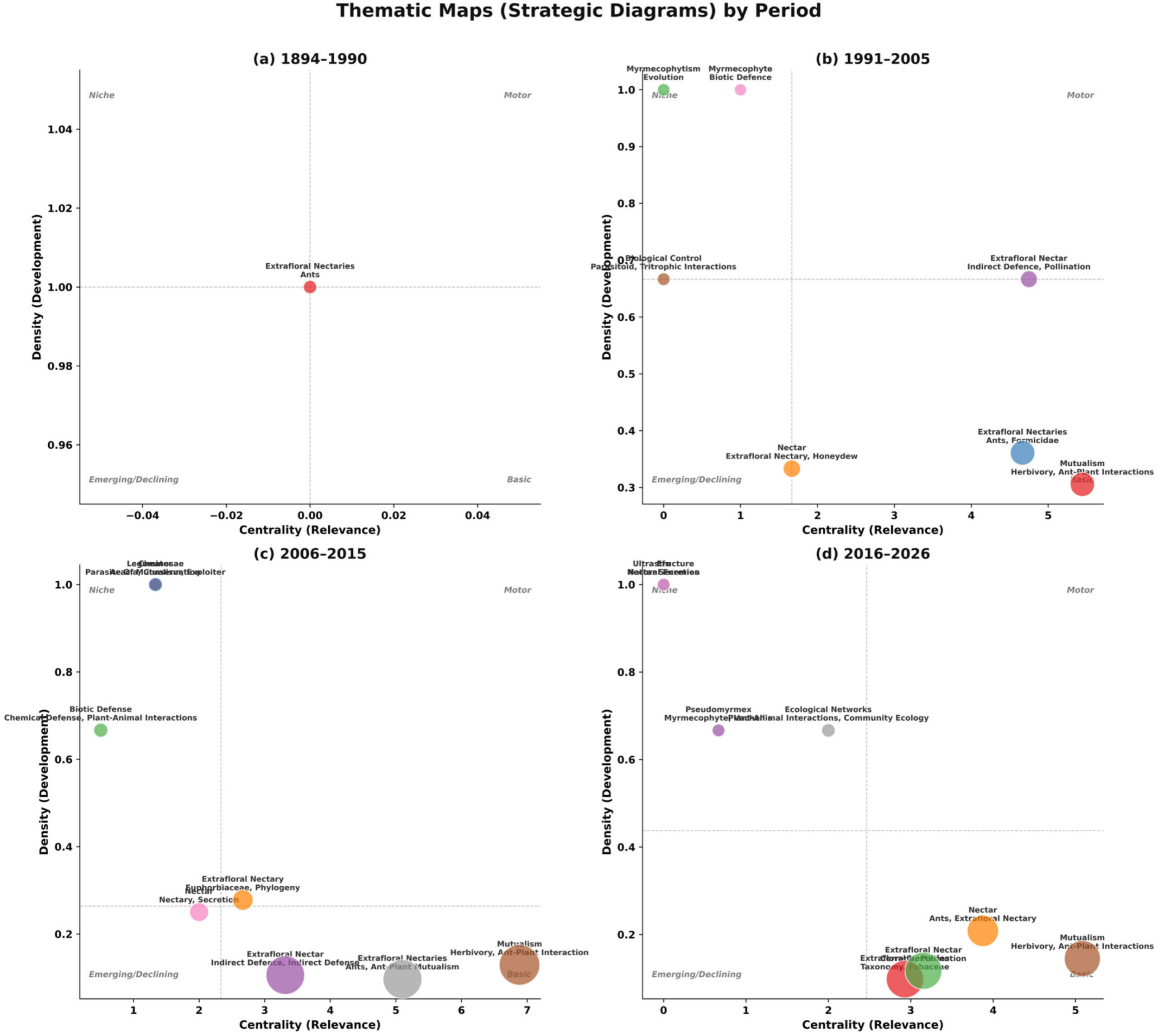
Thematic maps (strategic diagrams). Overall thematic map and period-specific maps for **(a)** 1894–1990, **(b)** 1991–2005, **(c)** 2006–2015, and **(d)** 2016–2026. Quadrants: Motor Themes (upper right), Niche Themes (upper left), Basic Themes (lower right), Emerging/Declining Themes (lower left).

The thematic map places keyword clusters along axes of centrality (relevance to the field) and density (internal development) (**Figure 9**). In the most recent period (2016–2026), the Motor Themes quadrant (high centrality, high density) contains extrafloral nectaries, mutualism, and herbivory, indicating that these topics are well developed and central to the field. Extrafloral nectar and indirect defense occur as basic themes (high centrality, low density). Multiple sub-communities, linked by these clusters but showing limited internal cohesion, functioning mainly as shared vocabulary rather than as integrated research programs. The Niche Themes quadrant (high density, low centrality) includes ant–plant mutualism with domatia and myrmecophyte, Cerrado-associated ecological networks, and nectary anatomy with secretory structures. These themes are methodologically cohesive and share citation cores but remain relatively isolated from mainstream EFN research. The Emerging/Declining Themes quadrant (low centrality, low density) contains climate change, ant–plant interaction, and facultative mutualism, suggesting topics that are either nascent or declining. Cross-referencing with thematic evolution and trend-topic analyses indicates that climate change is an emerging research front, first detected in 2016–2026 with a median keyword year of 2021, rather than a declining theme.

### 3.9 Thematic evolution

**Figure 10:**
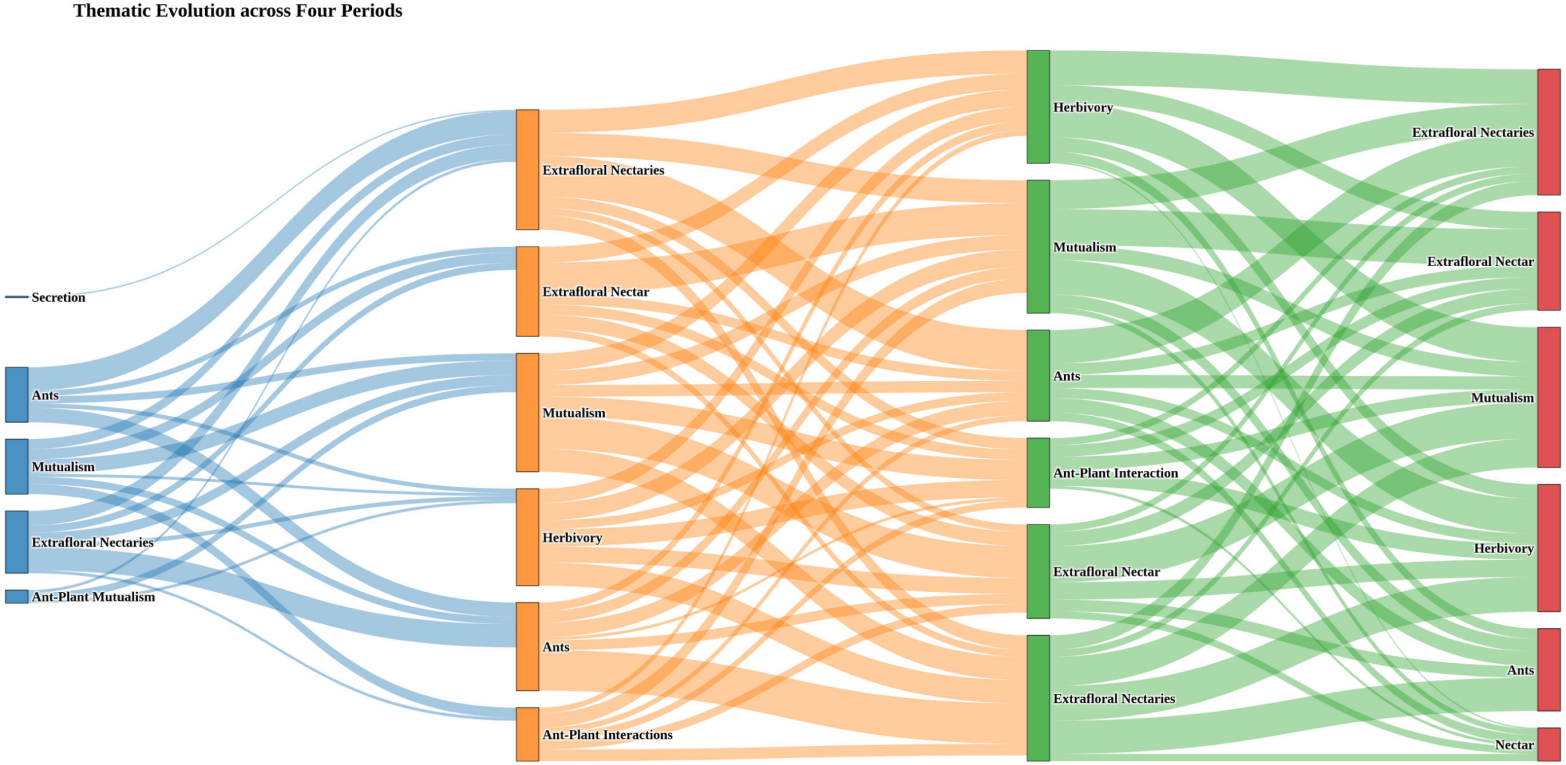
Thematic evolution of EFN research across four temporal periods (1894–1990, 1991–2005, 2006–2015, 2016–2026). Sankey bands trace thematic continuity, emergence, merging, and decline across periods.

The movement of thematic clusters across four temporal slices illustrates the development of EFN research over time (**Figure 10**).

Period 1 (1894–1990): This interval was mainly dominated by six themes: ant–plant mutualism, anatomy, nectary, extrafloral nectary, extrafloral nectaries, and mutualism. Research was largely descriptive, emphasizing EFN morphology, nectary classification, and documentation of ant visitation, mainly in tropical systems.

Period 2 (1991–2005): Thematic diversity increased. Extrafloral nectaries and nectaries persisted, and biological control emerged as a distinct cluster, indicating interest in EFN-bearing plants as habitats for parasitoids and predators in agroecosystems. Ant–plant interactions and extrafloral nectar also emerged as clear themes, indicating a shift toward chemical-ecological and network-ecological research areas.

Period 3 (2006–2015): In this period, Cerrado emerged as a major standalone theme, reflecting the expansion of Brazilian research on EFN ecology in savanna biomes. Extrafloral nectar remains as a distinct cluster, and ant–plant interactions broadened in scope. A longevity cluster developed, probably linked to studies on the temporal dynamics of nectar secretion and ant patrolling.

Period 4 (2016–2026): This period shows the highest thematic diversification. Extrafloral nectaries and extrafloral nectar remained core themes, while Cerrado continued to gain prominence. The concept of ant–plant mutualism was refined as distinct from broader ant–plant interactions, and nectar chemistry became more prominent. Climate change emerged as a recognizable thematic cluster, suggesting new work on how altered precipitation regimes and shifting phenologies reshape EFN-mediated mutualisms across biomes (Holopainen et al., 2020; Câmara et al., 2024). Trend-topic analysis shows rising keywords such as taxonomy (median 2020), biotic defense (2021), climate change (2021), facilitation (2022), Atlantic Forest (2022), and protective mutualism (2024), indicating wide research toward tri-trophic and community-level perspectives and an expansion into biomes beyond the Cerrado.

### 3.10 Collaboration networks

**Figure 11:**
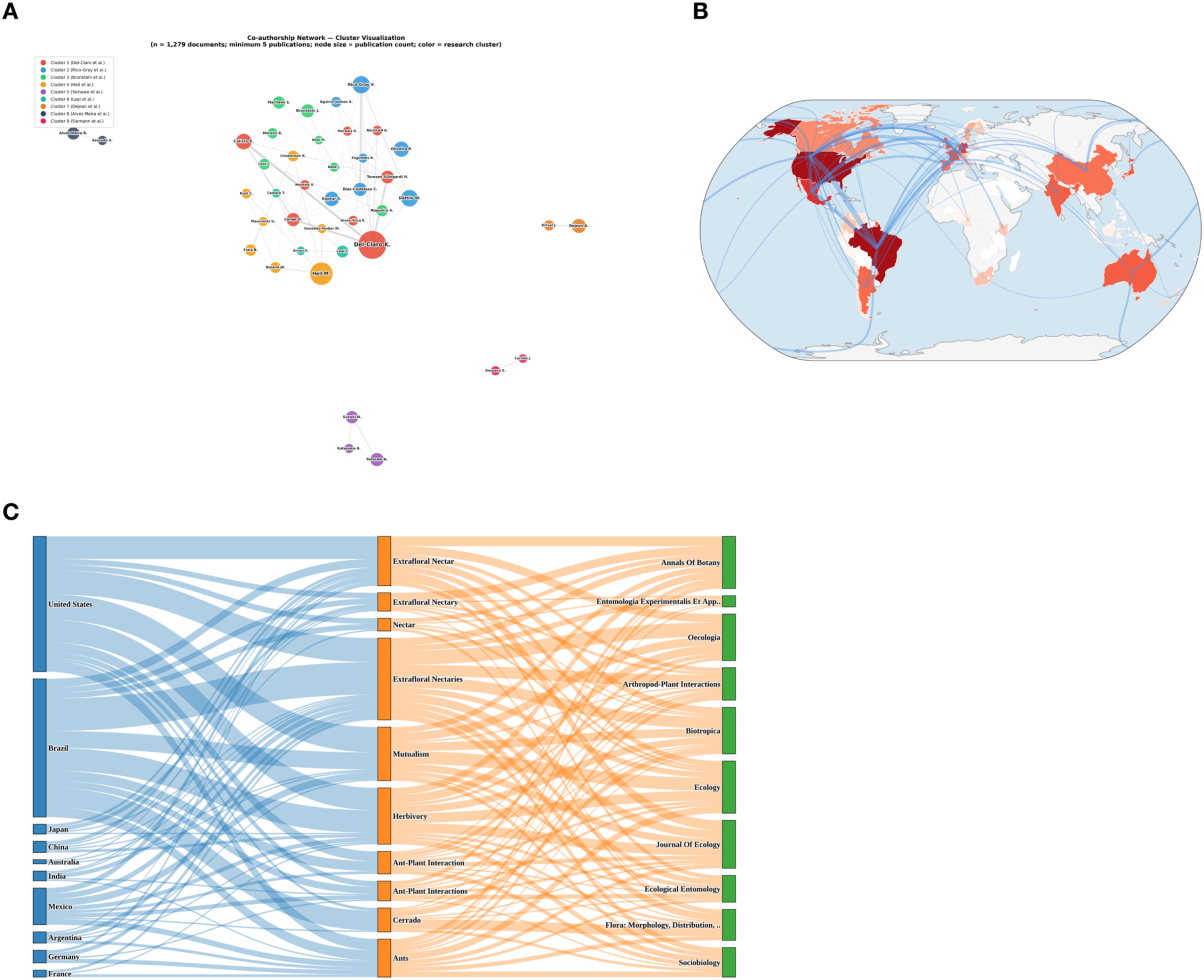
Collaboration analysis. **(A)** Author co-authorship network (Fruchterman-Reingold layout, top 30 authors, Walktrap clustering); **(B)** Country collaboration world map; **(C)** Three-field Sankey plot: Countries ↔ Author Keywords ↔ Journals.

Author-level co-authorship analysis delineated several discrete research groups (**Figure 11A**). The largest cluster centered on Del-Claro K, with strong collaborative ties to Calixto ES, Torezan-Silingardi HM, Lange D, Díaz-Castelazo C, and Alves-Silva EA, a predominantly Brazilian - Mexican group anchored in Cerrado and tropical-forest field systems. A second cluster, headed by Heil M, encompassed Boland W, Linsenmair KE, and Fiala B, researchers based primarily at the Max Planck Institute for Chemical Ecology, working on the molecular underpinnings of EFN induction. Rico-Gray V served as a bridge node linking Latin American and European collaboration networks. Koptur S led a largely independent cluster centered on Neotropical Island and subtropical EFN ecology, while Yamawo A represented a distinct Japanese network investigating EFN plasticity in temperate species.

The country-level collaboration map (**Figure 11B**) confirmed Brazil and the USA as the two global hubs, connected by the field’s strongest co-authorship corridor (56 joint papers). Mexico served as a critical tertiary node bridging Latin American and North American networks (33 links with Brazil; 23 with the USA). In transatlantic ties, European countries—Germany, France, Switzerland, and the Netherlands formed a secondary collaborative block. The three-field Sankey plot linking countries, author keywords, and journals (**Figure 11C**) revealed that Brazilian researchers channeled work on cerrado, ant-plant interactions, and mutualism primarily into Oecologia, Arthropod-Plant Interactions, and Sociobiology, while US-based authors contributed more broadly across Ecology, Journal of Ecology, and PLoS ONE.

## 4 Conclusions

This study presents the first bibliometric analysis of EFN research, covering 1,279 documents (1894–2026) from 343 sources by 2,660 authors across 51 corresponding-author countries. The field follows a logistic growth trajectory (K ≈ 1,460; R² = 0.938), with 87.6% of projected saturation reached. Brazil, the USA, and Mexico account for 44.5% of output. Five thematic clusters organize the literature, and thematic evolution traces a clear arc from morphological description to ecological experimentation to network-level analysis. Beyond these findings, the data expose four structural gaps that constrain the field’s trajectory.

### 4.1 The floral nectary imbalance

During PRISMA screening, 645 of 1,924 unique records (33.5%) were excluded because they addressed floral nectaries rather than EFNs, a ratio that quantifies the size disparity between these adjacent fields. Most of the molecular research is done on floral nectaries. The two key breakthroughs in nectary molecular biology, the SWEET9 sucrose transporter (Lin et al., 2014) and the CRABS CLAW transcription factor pathway (Lee et al., 2005), were achieved entirely in floral nectaries of *Arabidopsis* and *Nicotiana*. Kram and Carter (2009) noted that “virtually nothing is known about the molecular control of extrafloral nectary development.” Whether the CRC-dependent developmental pathway operates across the 457 independent EFN origins (Marazzi et al., 2013) remains one of the most fundamental unanswered questions in the developmental biology of EFN-bearing plants. The global investment in pollinator conservation following documented pollinator declines (Potts et al., 2010) has further concentrated funding on floral systems, contributing to the modest growth rate (1.83%) and saturation ceiling (K ≈ 1,460) observed here.

### 4.2 Geographic concentration

Research output was heavily concentrated in the Neotropics, with Brazil, the USA, and Mexico together accounting for 44.5% of corresponding-author articles. The presence of African nations was marginal. Southeast Asia, and the Indian subcontinent were also minimally represented. The gap is not explained by a scarcity of EFN-bearing taxa. Weber and Keeler (2013) documented EFNs across 129 families, many of which are species-rich in African and Asian biomes (Fabaceae, Euphorbiaceae, Combretaceae, Passifloraceae). Moles et al. (2011) demonstrated latitudinal variation in plant defense investment, suggesting unexplored EFN diversity across tropical Africa.

India (31 articles; MCP = 6.5%) and Japan (48 articles; MCP = 6.3%) present a specific pattern: substantial domestic output but minimal international collaboration. India has a long-established tradition of plant histochemistry and secretory tissue anatomy (Maheshwari, 1950; Fahn, 1979), but this capacity has been directed predominantly toward floral nectaries rather than EFN systems. The expertise to conduct EFN anatomical and histochemical research exists in these regions; it has simply not been mobilized for this purpose. The Brazil–Mexico collaboration corridor (52.9% MCP), the most internationally productive partnership in the dataset, offers a replicable model for building new South–South and North–South linkages that could extend EFN research into Africa, South Asia, and Southeast Asia (Confraria and Godinho, 2015).

### 4.3 Emerging frontiers: climate change and biological control

Climate change appeared as a thematic cluster only in 2016–2026 (median keyword year: 2021) and occupied the Emerging/Declining quadrant, novel but internally sparse. This is concerning because mutualistic interactions are disproportionately vulnerable to climatic disruption (Tylianakis et al., 2008), and EFN-mediated mutualisms depend on temperature- and moisture-sensitive processes at every trophic level: nectar secretion (González-Teuber and Heil, 2009), ant foraging activity (Câmara et al., 2024), and herbivore pressure. Whether the phenotypic plasticity of EFN secretion can buffer climate-driven changes faster than ant–plant partnerships decouple remains an open question requiring long-term field experiments across latitudinal gradients.

The biological control cluster (Cluster 3: 3 nodes) was the smallest and most isolated thematic group, despite strong empirical evidence for agricultural applications. Gurr et al. (2016) demonstrated across multiple Asian countries that engineering nectar resources into crop margins reduced pest populations by 70% and insecticide use by 70%. Khan et al. (2014) documented that push–pull companion-planting systems, which exploit nectar-based arthropod recruitment, improved food security for over one million smallholder farmers in East Africa. Koptur et al. (2015) showed that EFN-bearing cover crops increased parasitoid abundance in subtropical systems. Yet our data show this applied dimension has remained disconnected from mainstream EFN ecology, likely reflecting a disciplinary divide between fundamental ecologists and agricultural entomologists that needs to be actively bridged (Oliveira and Koptur, 2017).

### 4.4 Concluding remarks

The logistic growth model indicates that EFN research is approaching saturation under its current paradigm. The gaps identified here include the overshadowing effect of floral nectary research on molecular tools, the limited representation of Africa and South Asia in the collaboration network, a climate-change cluster that lacks internal cohesion, and an isolated biological control theme with demonstrated but unexploited agricultural potential. These define concrete opportunities to extend the field beyond its current trajectory. Capitalizing on these will require cross-continental partnerships, the systematic transfer of molecular tools from floral to extrafloral systems, and deliberate interdisciplinary integration between fundamental ecology and applied crop science.

### 4.5 Limitations

The geographic patterns reported are based on records indexed in Web of Science and Scopus and should not be read as a complete map of global EFN research effort. Although our search applied no language filter, both databases are known to predominantly index English-language journals and to under-index journals published in regional languages or based in Africa, Latin America, and parts of Asia (Mongeon and Paul-Hus, 2016; Vera-Baceta et al., 2019; Asubiaro et al., 2024). Books, book chapters, theses, and grey literature, which may carry a larger share of botanical research output in some regions are also under-represented in these indexes. EFN research published in non-English regional journals therefore cannot enter our dataset even though our inclusion criteria would have accepted it. Conclusions about underrepresented continents should therefore be read as referring to the EFN literature catalogued by these two databases, rather than as a claim about the absolute volume of EFN research conducted there. A targeted re-examination using regional indexes such as SciELO (Latin America), AJOL (Africa), and J-STAGE (Japan) is recommended for future work.

## Data Availability

All raw bibliographic data, the cleaned analytical dataset, the R reproducibility pipeline, Python figure-generation scripts, the record-level screening log, and the duplicates log supporting the conclusions of this article are openly available in the Zenodo repository at https://doi.org/10.5281/zenodo.20084533 under a Creative Commons Attribution 4.0 International (CC-BY 4.0) license.

## Footnotes

1 www.extrafloralnectaries.org
