# Supplementary material for: Mapping the research landscape of extrafloral nectaries: a comprehensive bibliometric analysis (1894–2026)
Source: Front Plant Sci. 2026 Jun 26;17:1848739. doi: 10.3389/fpls.2026.1848739 (PMC13350514; doi:10.3389/fpls.2026.1848739)

Supplementary Material

**Supplementary Figure S1.** Top authors' production over time in extrafloral nectary research. Bubble size indicates the number of articles published per year; color intensity reflects total citations per year (TC per Year).


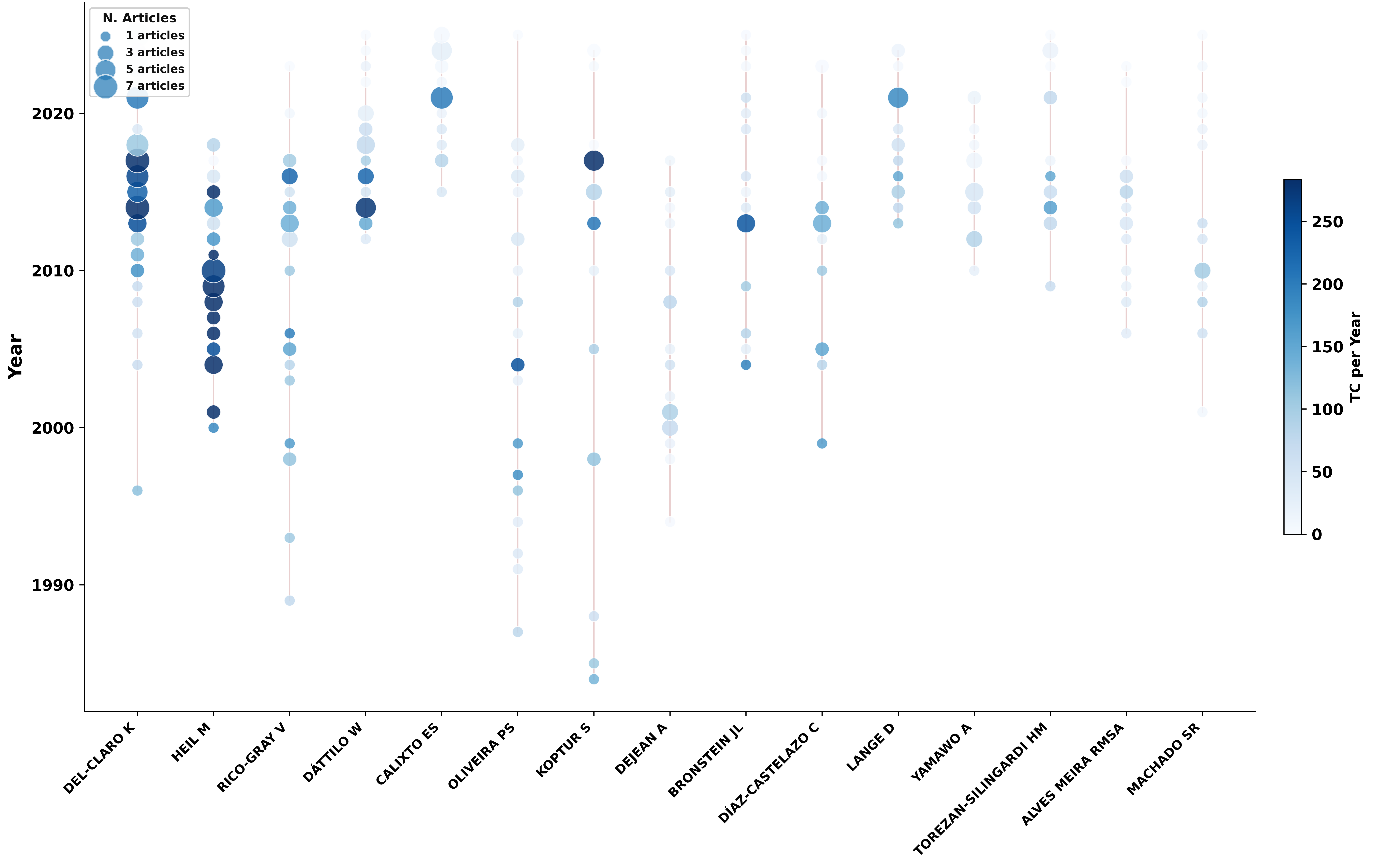


**Supplementary Figure S2.** Africa-involving collaboration network in extrafloral nectary research (1894–2026). Red nodes represent African countries (n = 10); blue nodes represent non-African collaboration partners. Node size is proportional to weighted degree. Edge thickness reflects collaboration frequency; numerical labels indicate joint paper counts (shown for ≥2). Blue edges denote cross-continental links; green edges denote intra-African links. The network comprises 49 bilateral links and 61 total joint papers.


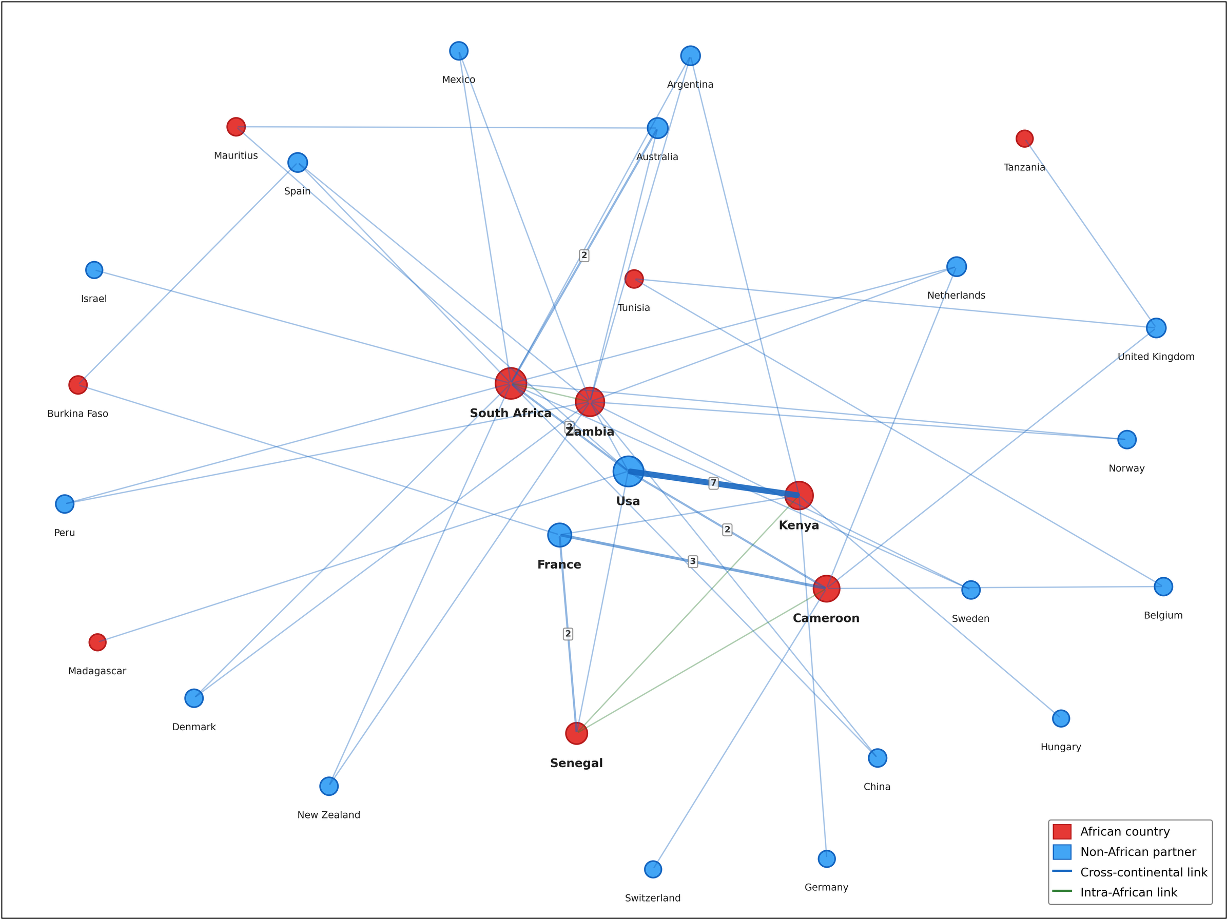

Supplement: Supplementary file 1 [file DataSheet1.docx]
